# Malaria Control with Transgenic Mosquitoes

**DOI:** 10.1371/journal.pmed.1000020

**Published:** 2009-02-10

**Authors:** John M Marshall, Charles E Taylor

## Abstract

John Marshall and Charles Taylor review recent advances in the development of transgenic mosquitoes for malaria control.

Malaria has been eliminated from a large part of the world. By the mid-twentieth century both North America and Europe were free of the disease, although both had suffered greatly during the prior century [1,2]. While a variety of means were used to achieve this eradication, the most important are thought to be reducing the number of breeding sites for malaria vectors and improving residential areas to separate humans from mosquitoes.

Other parts of the world have not been so fortunate. In sub-Saharan Africa, it is now estimated that there are more than 360 million clinical cases and one million deaths due to malaria each year [3,4]. Furthermore, despite ambitious goals such as those of the Roll Back Malaria Initiative to halve malaria deaths by 2010, mortality from the disease has actually risen halfway through the program [5]. Clearly the tools we have to control malaria, or the ways in which we are using them, are not working.

The failure of existing methods for malaria control has sparked interest in several new approaches. These include better and cheaper antimalarial drugs [6], renewed efforts to find a vaccine [7], and the development of genetically modified mosquitoes (GMMs) designed either to reduce population sizes or to replace existing populations with vectors unable to transmit the disease. In this review we describe some of the efforts currently underway to create GMMs and assess some of the obstacles they face.

## Background

Malaria in humans results from infection by any of five species of Plasmodium: P. falciparum, P. vivax, P. ovale, P. malariae, and P. knowlesi. These are transmitted to humans by approximately 50 species of mosquitoes, all belonging to the genus Anopheles. In sub-Saharan Africa, the vast majority of deaths are caused by P. falciparum transmitted by An. gambiae and the closely related An. arabiensis. These species are difficult to work with in the laboratory, so other model systems of malaria are often used in laboratory studies.

Most species of mosquitoes do not transmit malaria, and even among species that do, many individuals seem incapable of transmitting the disease, i.e., are refractory. Accordingly, there is reason to hope that the genes that permit malarial infections in mosquitoes can be identified and then replaced or altered in terms of their function. In this way, it is hoped that mosquito populations will become refractory to the parasite, eventually leading to malaria transmission being halted.

A variety of methods for engineering refractory mosquitoes are currently being studied and show promise for malaria control. The laboratory of Marcelo Jacobs-Lorena at Johns Hopkins University has successfully engineered mosquitoes that confer resistance to rodent malaria [8]. Their approach was to first identify receptor sites for proteins that the parasite requires to pass through the gut after ingestion. They next produced small proteins that saturate the receptor sites and hence block amplification and transmission of the parasite (Figure 1). Future research in this area should focus on optimizing refractory genes to effectively confer resistance to human malaria.

**Figure 1:**
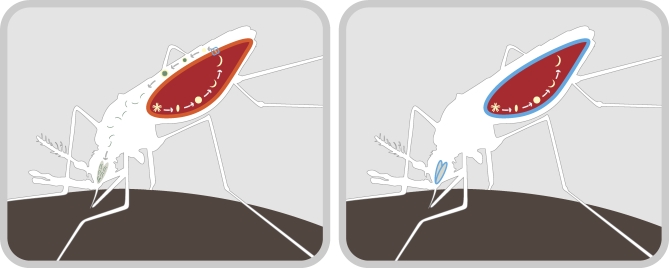
Mechanism for Blocking Malaria Transmission in the Mosquito Left: Mosquitoes become infected with the malaria parasite upon taking an infected human blood-meal. This produces an oocyst in the mosquito's gut wall (light red). When the oocyst ruptures, it releases sporozoites that pass through the gut (dark red) and into the hemocoel (white). The sporozoites are then amplified and migrate through the mosquito's body to the salivary glands, ready to infect a new human. Right: The laboratory of Marcelo Jacobs-Lorena at Johns Hopkins University has identified receptor sites for proteins that are necessary for the malaria parasite to pass through the gut wall after the oocyst ruptures. The same receptors are involved with the passage of sporozoites into the salivary glands. The laboratory has produced small proteins that preferentially occupy these sites (blue), blocking transmission of sporozoites through the gut wall and into the salivary glands. The appropriate gene constructs have been introduced into An. stephensi mosquitoes, thus rendering them refractory to P. berghei (a model system for human malaria).

Other methods for generating refractoriness involve using antibodies that kill parasites within the mosquito [9] and discovering genes that govern refractoriness in natural populations [10]. A great deal is being discovered about the immune system of mosquitoes [11], leading many researchers in this field to believe that an effective gene construct to reduce the ability of mosquitoes to transmit malaria is not far away.

**FIVE KEY PAPERS IN THE FIELD**

**Marshall, 2008** [18] This article focuses on TEs as a drive system and models the impact of dissociation between the drive system and refractory gene. It references much of the important work to this time on TEs.

**Chen et al., 2007** [20] This article describes the biology and potential uses of a synthetic *Medea* element observed to spread through laboratory Drosophila populations.

**James, 2005** [19] A general overview of the criteria required by gene drive systems intended to drive refractory genes into mosquito populations.

**Alphey et al., 2002** [27] A discussion of the benefits, risks, and research priorities associated with using transgenic insects to control vector-borne diseases.

**Ito et al., 2002** [8] An historic paper that describes one of the first candidate antiparasitic genes that works in a disease vector model system in the laboratory.

## Drive Systems

More problematic is the means of driving a refractory construct quickly and efficiently through the vector mosquito population so that the population of susceptible mosquitoes will be replaced. Transposable elements (TEs) were one of the first gene drive systems to gain widespread attention for population replacement [12]. These elements are able to spread quickly through a population due to their ability to replicate within a host genome and hence to be inherited more frequently in the offspring's genome. This increase in inheritance enables TEs to spread even in the presence of a fitness cost to the host [13]. It has also led to their widespread prevalence among many taxa, to the extent that various families of TEs represent 47% of the Aedes aegypti mosquito genome [14].

One source of encouragement for the use of TEs in population replacement is the observation that the *P* element spread through most of the global Drosophila melanogaster population within the span of a few decades following a natural acquisition from D. willistoni [15]. It is hoped that such an invasion could be repeated in a mosquito species using a TE that is attached to a refractory gene conferring resistance to malaria. Ideally, such an invasion would be repeated in each of the major mosquito species that transmits malaria.

Despite initial excitement, TEs have become less favored as a means of population replacement in recent years. The first major hurdle has been the failure to introduce a highly active TE into An. gambiae—the main vector of malaria in sub-Saharan Africa. TEs tend to repress their activity over time to avoid corrupting the host genome. Many TEs, including the *P* element, accumulate mutations leading to their inactivation. This may make the discovery of a highly active TE more challenging than originally anticipated.

Additionally, preliminary data suggest other reasons that TEs may be ill-suited to driving foreign DNA into populations. A study on the *Himar1 mariner* element suggests that TE activity declines substantially with increasing size [16]. Given current refractory gene sizes (e.g., [17]), the *mariner* element is estimated to have its replication rate reduced by at least 95% when burdened by a refractory construct [16]. Its drive would have to be very strong in order to suffer such a decline in replication.

This is compounded by the fact that TEs are particularly vulnerable to losing internal sequences during replication. Mathematical modeling suggests that, if the refractory gene is lost from the TE at a modest rate, the malaria-susceptible TE will return to again dominate the population [18]. Therefore, even if active TEs can be identified, their ability to drive refractory genes into a population is questionable.

Disenchantment with TEs as a means of population replacement has coincided with interest in several other gene drive systems. Some of the most promising drive mechanisms currently being investigated include *Medea* elements, homing endonuclease genes (HEGs), engineered underdominance constructs, and the intracellular bacterium Wolbachia. Other systems that are being investigated include engineered underdominance constructs and meiotic drive [19].

The favorability of one gene drive system over another will depend on its ability to quickly and efficiently spread a refractory gene. However, this on its own is not enough. The ideal gene drive system will also address ecological, epidemiological, and social concerns that such a system engenders and minimize the likelihood of any risks. In our opinion, the most promising system at present is *Medea*.

*Medea* has attracted much attention as a tool for population replacement in recent years, following the observation that an engineered *Medea* element is able to rapidly spread through D. melanogaster populations in the laboratory [20]. The design of this synthetic element is based on a naturally occurring selfish genetic element first discovered in a species of flour beetle, Tribolium castaneum. *Medea* is able to rapidly spread through a population in the presence of a fitness cost by distorting the offspring ratio in its favor. It does this by causing the death of all offspring of heterozygous females that do not inherit the allele, thus giving rise to its name—an acronym for maternal-effect dominant embryonic arrest, with reference to the mythological Greek figure who murdered her own children.

The synthetic *Medea* element developed by Chen et al. [20] works by the hypothesis that *Medea* encodes both a maternally expressed toxin and a zygotically expressed antidote. The toxin causes the death of all progeny lacking the *Medea* allele, and the antidote rescues *Medea*-bearing progeny from an otherwise imminent death (Figure 2). In this way, the proportion of *Medea*-bearing individuals is increased with each generation; and it is hoped that an attached refractory gene conferring resistance to malaria could come along for the ride.

**Figure 2:**
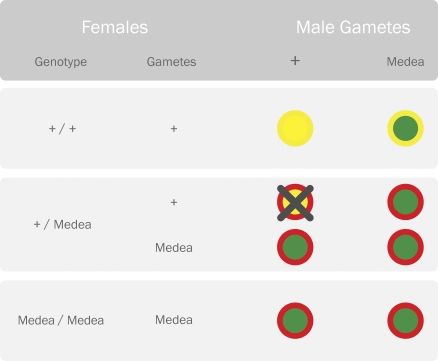
Parental Crosses Representing the Reproductive Advantage of the *Medea* Allele Females carrying the *Medea* allele produce a maternally expressed toxin (red outer circle) that is deleterious to their offspring. Offspring who carry the *Medea* allele are rescued by a zygotically expressed antidote (green inner circle) expressed by the same allele. Offspring of heterozygous females who do not inherit the *Medea* allele are killed by the toxin because they lack the antidote (yellow represents lack of the toxin/antidote). This distorts the offspring ratio in favor of the *Medea* allele.

*Medea* does not suffer from many of the ailments inflicted upon TEs—an active *Medea* element has been engineered, its spread is not retarded by the insertion of foreign DNA, and a solution has been proposed to minimize the rate of dissociation of refractory genes [20]. Additionally, in the event that a refractory gene should be recalled following an environmental release with unwanted consequences, it has been proposed that another strain of *Medea* could be introduced to replace the first, thus removing the refractory gene from the population.

One attractive feature of *Medea* is that its rate of spread is strongly dependent on its release ratio [21]. While *Medea* will spread very quickly following a large intentional release, it is very likely to go extinct following a small accidental release [22]. This is particularly important since it is impossible to guarantee that there will be no escapes while outdoor cage trials assess the potential outcomes of an environmental release [23]. *Medea* therefore presents a desirable balance between invasiveness and containment. At present, there is an active effort to construct *Medea* systems for mosquitoes, but as yet no such systems have been made.

HEGs are another system for which there are active development efforts. These genes are able to spread through a population by expressing an endonuclease that creates a double-stranded break at a highly specific site lacking the HEG. Homologous DNA repair then copies the HEG to the cut chromosome, thus increasing its representation over subsequent generations [24].

## Next Steps in Research

The first requirement of any transgenic mosquito project will be the discovery of genes that confer resistance to human vector-borne diseases. The proof of principle has been shown for rodent and chicken malaria, and it remains to optimize genes to confer resistance to human malaria. Several refractory genes will be necessary for a successful intervention both to improve the efficacy of refractoriness, and to reduce the probability that resistance to antipathogen genes will emerge in the Plasmodium population.

Possibly more challenging will be the optimization of gene drive systems to deliver these refractory genes into mosquito populations. *Medea* has been shown to drive population replacement in Drosophila; and future research should work towards repeating this in mosquitoes. If this can be achieved, *Medea* will be a very promising candidate for population replacement; however, potential hazards for *Medea* and other gene drive systems must be identified and responded to, such as their ability to spread through reproductively isolated populations, and their persistence following an accidental release. Mathematical modeling can assist in assessing the severity of these concerns.

A broad study is required of the ecology of mosquito vectors through which the refractory genes are intended to be driven. Comprehensive ecological studies have been carried out in selected regions (e.g., [25]); however, these must be extended to other regions of Africa to gain a broader picture of species distributions and rates of gene flow. Malaria is a complex disease, and the biology of its vectors is also complex. In most parts of Africa, there is more than one species of Anopheles that transmits malaria. If hybridization among species is judged to be insufficient, then the feasibility of altering several species of malaria vectors will need to be considered.

We have focused our review on the effort to produce GMMs for malaria control; however, developing GMMs for dengue control will likely be achieved much earlier. Dengue virus, transmitted by the vector Ae. aegypti, is likely the second-most important vector-borne disease system after malaria. It is also much simpler than malaria—Ae. aegypti is easier to rear and experiment with than An. gambiae, and dengue does not have a complicated life cycle like Plasmodium. Much of the current work on GMMs is being conducted with dengue virus, and many of the problems confronting vector replacement will probably be worked out first with this system.

Finally, a large number of ethical concerns must be addressed and resolved satisfactorily before GMMs can be introduced. These include questions about the meaning of informed consent in communities that are largely illiterate, unfamiliar with genetic modification, and sometimes uneducated on the role of mosquitoes in disease transmission. These consent issues are confounded by the possibility of unknown and potentially serious side effects of a release, for example, an increase in the transmission of non-target diseases. Furthermore, acceptance by one community, or even country, is likely to affect many of its neighbors, whether they agree with the decision to release or not. Such a release may occur accidentally from an outdoor cage trial; however, an intentional release cannot be conducted prior to evaluation in cage trials.

Despite this, mosquito-borne diseases kill in excess of a million people every year, mostly children under five years old. GMMs offer some hope of reducing this burden of disease, and hence their risks, both known and unknown, must be weighed against the certain toll of inaction. In addition to some helpful initial studies [22,26,27], there is a clear need for much more analysis of the human research participant issues posed by these new methods.

**GLOSSARY**

**Refractory gene:** Gene conferring inability to transmit malaria.

**Transposable elements:** Genomic components that express a transposase enzyme catalyzing their replication in the genome. This enables them to spread through a population despite a fitness cost.

**Fitness costs:** Reduction in fitness associated with carrying foreign DNA.

***Himar1 mariner element*:** A transposable element of the *Mariner* class. It contains its own transposase gene and moves by a cut-and-paste mechanism.

**Internal sequences:** Region of DNA between the characteristic end sequences of a transposable element.

**Homing endonuclease gene:** A selfish gene that spreads through a population by expressing an endonuclease that creates a double-stranded break in a DNA sequence and then copies itself to this site.

***Medea* element:** A selfish genetic element that is able to spread through a population through its ability to cause the death of all offspring of heterozygous females that do not inherit the allele.

**Wolbachia:** A maternally inherited intracellular bacterium that can disrupt reproduction with noninfected sperm. This may be used to drive refractory genes into vector populations.

**Engineered underdominance constructs:** A form of underdominance in which there are two transgenic constructs, each of which possess a lethal gene and a suppressor gene that down-regulates the expression of the lethal gene on the other construct.

**Meiotic drive:** Any mechanism by which a heterozygous locus segregates at a greater-than-Mendelian frequency, often by destroying or disabling the homologous chromosome.

**Dissociation of refractory genes:** Loss of refractory gene DNA from a gene drive construct such that the refractory gene no longer functions.

**Release ratio:** The ratio of transgenic mosquitoes to natives ones at the time of a release.

## Conclusion

Malaria control with transgenic mosquitoes will be challenging; however, recent advances suggest that it may be a possibility in the foreseeable future. Progress towards discovering refractory genes for rodent malaria and gene drive systems for Drosophila provide hope that similar advances may be made for human malaria in mosquito vector species.

That said, the African malaria burden has proved exceptionally difficult to diminish by all means tried thus far; and it is unlikely that transgenic mosquitoes will provide an all-in-one solution. Transgenic mosquitoes should be considered within the context of an integrated vector management strategy which should also include insecticide-treated bed-nets, indoor residual spraying with insecticides, and treatment of infected individuals with antimalarial drugs. Integrated strategies will be a necessity for any successful African malaria control program [28]; and transgenic mosquitoes should be considered as a potential ingredient in the future goal of continent-wide disease control.

## Abbreviations

GMM: genetically modified mosquito
HEG: homing endonuclease gene
TE: transposable element
